# New strategies to slow down the photoaging of the human skin

**DOI:** 10.1186/1878-5085-5-S1-A147

**Published:** 2014-02-11

**Authors:** John Ionescu

**Affiliations:** 1 Spezialklinik Neukirchen, Germany and Donau University Krems, Austria

## 

Previous reports indicate that inflammatory and proliferating skin conditions like acne and psoriasis are also associated with decreased ATP and cyclic nucleotides (cAMP) in blood and epidermal cells. In the late 80’s we noticed that psoriatic skin lesions significantly respond to supplementation with energy generating compounds and melanin promoters. In clinical studies, topically applied energy generating compounds like AMP, ADP and NAD normalized the cell replication rate in psoriatic skin, (CELL ENERGY) diminished the acne pustulae and induced a significant improvement of skin structure and wrinkles (anti-aging-effect). The photoaging process of the human skin in the presence of natural sunlight or artificial UV-sources is closelyy related to a strong generation of free radicals, lipid peroxidation, collagenase activation, glycation/ oxidation of proteins (AGE products), activation of p53 transcription factors, low DNA repair capacity and cumulative DNA mutations. Clinically, the adverse effects of natural sunlight and other UV-sources on normal human skin may vary from sunburn with erythema, oedema and DNA damage (12-24 hrs. after UV-exposure) to polymorphic light reaction (eczema solare), solar actinic elastosis and actinic hyperkeratosis (as common precancerous condition), up to different skin cancer forms like basal cell carcinoma (BCC), squamous cell carcinoma (SCC) or malignant melanoma (MM). The study of the lipid, protein and DNA oxidative damage triggered by the free radical attack subsequent to sunlight exposure has conducted to appropriate strategies to slow down or block these reactions making possible the design of innovative skin care formulations. In this respect, a new German photoaging defence formula (SOLARIS SPF 25) combines for the first time the double UVA + UVB protection (SPF 25) with melanin promoting aminoacids directly enhancing the natural tanning process. Free radical blocking agents like Vit. E, carrot oil and metal chelators are preventing the sun exposure side-effects together with immune stimulating plant extracts (β-glucans) with anti-herpes virus activity. To slow down the photoaging related wrinkle formation efficiently, a new collagen synthase stimulating formula offers a synergistic anti-aging combination of UV-light blockers, free radical quenchers (Vit. E, Coenzyme Q10) and collagen/ elastin synthesis promoters like hydroxyprolin and plant bioflavonoids (ENERGO Repair)). The active ingredients are incorporated in liposomes containing skin identical phospholipids and ceramides by means of the patented DMS^®^ nanoparticle technology. A rapid uptake in the epidermis cells is thus granted. The use of the described hypoallergenic topical products results in a significant improvement of the skin structure and appearance within 30 days, as documented with the standardized Surface Evaluation of Living Skin (SELS) methodology in a group of 35 women aged 40 to 63 years.

Our recent research shows that appropriate combinations of hypoallergenic protein hydrolysates with sugar alcohols and omega-3 polyunsaturated fatty acids (EQUIDERM PLUS) are showing a remarkable free radical quenching and anti-inflammatory activity leading to a significant symptom alleviation in skin and geriatric patients. Finally, with the emergence of affordable microarray gene expression profiling methods we had the opportunity to test several oral and i.v. nutraceutical combinations (NUTRIGEN PA, NUTRIGEN PS ) for their silencing ability on inflammatory genes (nutrigenomic evaluation) and correlate the “ex in vivo” results in blood cells with their “ in vivo” clinical efficacy.

